# Safety and Efficacy of Thrombolytic Therapy Using rt-PA (Alteplase) in Acute Ischemic Stroke

**DOI:** 10.5402/2011/618624

**Published:** 2011-11-29

**Authors:** Palanisamy Sivanandy, Binny Thomas, Vijayan Krishnan, Sumathy Arunachalam

**Affiliations:** ^1^Department of Pharmacy Practice, KMCH College of Pharmacy, Kovai Estate, Kalapatti Road, Coimbatore, Tamilnadu 48, India; ^2^Department of Neurology, Kovai Medical Center and Hospital, Coimbatore, Tamilnadu 44, India; ^3^Department of Pharmacy, Madurai Medical College, Madurai, Tamilnadu 20, India

## Abstract

The aim of this study was to evaluate the efficacy and safety of thrombolytic therapy. Our study enrolled 23 patients out of which 2 patients died due to ICH; we also found that only 39% of patients reached hospital within stroke window, and for those treated, mean NIHSS during admission was 14.0, but drastic improvement was shown after 24 hours of treatment, that is, 9.89 (*P* < 0.0001), and at discharge, it was 5.1 (*P* < 0.0001) which showed a clear impact of the treatment, and around 60% of patients were discharged within an mRS score of 1 and 2. Hence, it was found that thrombolytic therapy was beneficial, efficacious, and safe if used within 3 to 4.5 hours.

## 1. Introduction

Cerebrovascular disease usually occurs in the middle and late stages of life; the incidence of stroke increases with age; and thus disability occurs to many people in their “Golden Years.” Some types of the cerebrovascular diseases include ischemia infarction and intracranial hemorrhage. Most cerebrovascular diseases are manifest by abrupt onset of focal neurological deficit. The deficit may remain fixed or may rapidly improve or progressively worsen. It is this abrupt onset of nonconvulsive and focal neurological deficit that defines stroke, or cerebrovascular accident. Reduction in cerebral blood flow causes cerebral ischemia which may last for seconds to minutes; neurological symptoms can be seen within few seconds because neurons lack rapid energy failure, and if the blood flow is recovered soon, the symptoms are only transient and thus termed as transient ischemic attack (TIA). It becomes very important for a clinician to always identify, differentiate, and then treat a stroke; they should differentiate ischemia infarction and hemorrhage since their treatment depends on the cause; the main focus is on two goals such as to prevent or reverse acute brain injury and to prevent future neurologic injury [1].

A rapid evaluation is essential for the time-sensitive treatments such as thrombolysis; there are several other causes of the sudden onset of neurological symptoms that may mimic stroke which are seizures, intracranial tumors, migraine, and metabolic encephalopathy. So we have to differentiate all these and then diagnose a stroke; once the diagnosis is made, a brain imaging study is essential to find the exact cause of the stroke and whether the type of stroke is ischemic or hemorrhagic. CT imaging becomes the standard imaging modality to detect the presence or absence of intracranial hemorrhage. If the stroke is ischemic and the patient is reaching hospital within a time period of 3 to 4.5 hours, she is a right candidate for thrombolysis using recombinant tissue plasminogen activator, and then medical management to reduce the risk of complication becomes the next priority followed by plans for secondary prevention [2].

An acute occlusion of an intracranial vessel causes reduction in blood flow in the region it supplies. A fall in cerebral blood flow to zero causes death of brain tissues within 4 to 10 minutes; values <16 to 18 mL/100 gm of tissues per minute cause infarction within an hour, and values less than 20 mL/100 gm cause ischemia without an infraction unless prolonged for several hours or days. Tissue surrounding the core region of infarction is ischemia but reversibly dysfunctional and is referred to as ischemic penumbra.

### 1.1. Treatment of Acute Ischemic Stroke

After the clinical investigation and diagnosis of stroke is made, it becomes very necessary to have an orderly process of evaluation, and treatment should be done. The first goal should be to prevent and reverse the brain injury, then attend to his airways, vitals, and breathing, and treat hyper- or hypoglycemia if any. We should always perform a noncontrast head CT scan to rule out any bleed. The treatment mainly falls within 6 categories, namely, medical support, intravenous thrombolysis, endovascular techniques, antithrombotic treatments, neuroprotection, and stroke centers and rehabilitations [3].

 Even after many studies have proven the safety and efficacy of thrombolytic therapy using Alteplase, very few centers use this method and treat the patients, and several reasons explain this situation: a limited therapeutic window, insufficient public knowledge of warning signs for stroke, a smaller number of centers available with facilities for the method, an excessive fear of hemorrhagic complications. In fact, rt-PA is the only drug licensed for treatment of acute ischemic stroke [4].

### 1.2. Thrombolysis and Alteplase

Thrombolysis is mainly caused by a class of drugs called fibrinolytic drugs, and this class of drugs break the thrombi by catalyzing the formation of serine protease plasmin from its precursors zymogens, plasminogen. These drugs cause lysis of clot when administered intravenously. Thus, both protective haemostatic thrombi and target thromboemboli are broken down. Plasminogen can also be activated by tissue plasminogen activator. They activate plasminogen that is bound to fibrin which causes fibrinolysis of the formed thrombus and finally avoids the systemic activation. Human t-PA is manufactured as Alteplase by means of recombinant DNA technology. This drug produces its effect by initiating local fibrinolysis by binding to fibrin in a thrombus (clot) and converts entrapped plasminogen to plasmin.

### 1.3. Pharmacodynamics and Pharmacokinetics

#### 1.3.1. Durations

>50% present in plasma is cleared in 5 minutes, and after infusion terminated, 80% is cleared within 10 minutes.

#### 1.3.2. Excretion

Rapidly from circulating plasma (550 to 650 mL/min), primarily hepatic, >50% present in plasma is cleared within 5 minutes, and after the infusion is terminated, 80% is cleared within 10 minutes.

#### 1.3.3. Dosage

Doses should be given within 3 to 4.5 hours after the onset of symptoms. Recommended total dose is 0.9 mg/kg and maximum dose is 90 mg.

### 1.4. Monitoring Parameters

In acute ischemic stroke, in addition to monitoring for bleeding complications, the 2007 AHA/ASA guidelines for the early management of acute ischemic stroke recommend the following:

1. perform neurological assessment every 15 minutes during the infusion and every half an hour thereafter for the next 6 hours then hourly until 24 hours after the treatment;
2. if severe headache, nausea, acute hypertension, vomiting occur, then discontinue the infusion and do a CT scan;
3. monitor BP every 15 minutes for the first 2 hours and then every 30 minutes for the next 6 hours, then hourly until next 24 hours after the initiation of Alteplase;
4. obtain follow-up CT after 24 hours before starting anticoagulants and antiplatelets.

### 1.5. Adverse Drug Reaction

Bleeding, hemorrhage, may occur at any site, reperfusion-related atrial or ventricular arrhythmia. Some others are hypotension, fever, bruising, GI bleed, nausea, and vomiting, all these to a lesser extent [5]. Thrombolytic therapy for stroke was reported for the first time in 1958, and then a trial was done in 1963 [6].

### 1.6. The National Institute of Health Stroke Scale (NIHSS)

We can assess the patient and select the suitable candidates based on a scale for physical examination; for thrombolytic therapy, a patient must have more than a minimal neurological deficit. Patients with only minimal weakness, isolated ataxia, isolated sensory deficit, or isolated dysarthria are generally not candidates to be thrombolysed. NIHSS is a scale used for neurological examination of the patient showing a level of consciousness, gaze difficulty, facial weakness, limb weakness, sensory loss, and speech problems. Scores range from 0 to 42. FDA applies that scores beyond 22 are of a severe neurological deficit. And patients are more prone to intracranial hemorrhage if they are administered with rt-PA after a score of 22. However, patients with NIHSS more than 22 still benefit from the treatment [7].

### 1.7. Modified Rankin Scale (mRS)

We also have a scoring system to measure the outcome events of the patients, for this, we use modified Rankin scale; it starts with 0 (no disability) and ends with 6 (death), and the primary measure of outcome is the proportion of patients alive and independent (mRS 0–2).

In our hospital, we are performing thrombolysis for acute ischemic stroke under the Department of Neurology, using a drug called Alteplase (a recombinant tissue plasminogen activator); this subject seemed to be too keen for me to study its efficacy and to analyze and report on the safety of this wonder drug, and this made us study Alteplase; if this treatment is successful and safe, we can also create awareness in the crowd about stroke and its treatment, within a thrombolytic time window (3 to 4.5 hours), and we can generate awareness of the importance of reaching hospital as soon as possible. Our aim is to report on the safety and efficacy of the treatment, to what extent the drug is safe to use, and how effective is the treatment. We have different scales to assess the outcome of the therapy; using these scales, we can come to a conclusion about the efficacy of the drug.

## 2. Methodology

### 2.1. Study Site

The study was conducted in a private corporate hospital which has all facilities under one roof. It is a 700-bedded multispecialty hospital having a wide range of specialties such as neurology, radiology, nephrology, urology, oncology, general medicine, diabetology, surgery, obstetrics, gynecology, cardiology, cardiothoracic surgery, pulmonology, orthopedics, ophthalmology, dentistry, ENT, and physical medicine. Neurology and Radiology Departments of the hospital were particularly chosen for the study, which has enormous potential for diagnosing and treating acute ischemic stroke.

### 2.2. Study Period

The study was planned to be carried out for a period of nine months.

### 2.3. Study Design: Retrospective and Prospective Study

The protocol of the study which includes the objectives and methodology was submitted to the Chairman, KMCRET & KMCH. The authorization of the Chairman and Medical Director was obtained to carry out the study.

### 2.4. Inclusion Criteria

Inpatients with above 18 years of age, those who were admitted in the hospital for treatment of acute ischemic stroke and were infused with Alteplase, were included in this study. Patients with no history of bleed, CT clearly diagnosing acute ischemic stroke, no recent surgeries, and onset of symptoms less than 3 hrs were included.

### 2.5. Exclusion Criteria

Patients with unconscious state, NIH score <4 and >25, pregnancy, seizure onset, bleeding disorders, and platelet count <100 and patients reaching hospital after thrombolytic time window period were excluded from the study.

## 3. Results and Discussion

The prospective and retrospective study has shown that the incidence of the stroke is more common in men than in women; 95.6 (*n* = 22) percentage of the men and 4.34 (*n* = 1) percentage of the women were admitted with stroke and thrombolysed.

The agewise classification revealed that most of the study population were elderly with the age group between 61 and 80 (*n* = 11), followed by the age groups 41 to 60 (*n* = 9), 21 to 40 (*n* = 2), and above 80 (*n* = 1); no patient was thrombolysed in the age group between 0 and 20. This indicates that elderly patients were in need of thrombolytic therapy.

In this study, 95.6 (*n* = 22) percentage of the study populations are married, and 4.34 (*n* = 1) percentage are single; this indicates that married people are more prone to have stroke than unmarried, and this may be due to change in food habits, life style, stress, and other factors.

This study says that only 7 (34.78%) patients were smoker and 6 (26.08%) patients were both smoker and alcoholic. 9 (39.14%) among 23 of the study population were not having any habits of smoking or alcohol. This report is in contrast with other studies, and this may be due to race, ethnicity, or any other factors which may concern with the disease.

The patient's medical history revealed that 21.73% (*n* = 5) of the study population had hypertension and 21.73% (*n* = 5) had both hypertension and diabetes, which is followed by coronary heart disease and hypertension (8.69%; *n* = 2), diabetes alone (8.69%; *n* = 2), and it is followed by 4.34% (*n* = 1) each in seizure + hypertension + urinary tract infection, diabetes and stroke, diabetes and coronary heart disease, hypertension + stroke, hypertension + diabetes + stroke, and hypertension + respiratory tract infections. In this study population, around 13% (*n* = 3) of the patients do not have any previous history of disease(s), and they experienced stroke for the first time.

The past medication history of the study population has shown that nearly 21.73 percent of the study population received antihypertensive with antidiabetics as past medication, 17.39 percent received antianginal and antihypertensive, and the same percent of study population did not receive any drug before getting admission into the hospital. 13.04 percent received antihypertensive as monotherapy, and the same percent received the antihypertensive with lipid-lowering agents as combination therapy. Only the least number (*n* = 1) of patients received antidiabetics and antianginal and antihypertensive + antiepileptics + antibiotics as combination therapy. This data revealed that most of the study population were diabetic and hypertensive, which are the risk factors for stroke (Table 1).

In this study population, the left middle cerebral arteries were affected more in around 60.86% of the study population, and right middle cerebral arteries were affected in around 13.04% of patients; both intracranial artery and middle cerebral arteries were affected in 8.6%, and intracranial artery alone was affected in 8.6%; intracranial artery, middle cerebral artery, and occipital lobe were affected in 4.3%, and basilar artery was affected in 4.3% of the study population. This revealed that most of the patients who had stroke were affected with left middle cerebral arteries (Table 2). 

The past history of stroke concluded that 83 percentage of the study population previously had stroke, and only 17 percent had the stroke for the first time. This indicates that the stroke patients are more prone to have recurrent attack and alarms the physician and patients regard the severity and the need of effective treatment.

**Figure 1:**
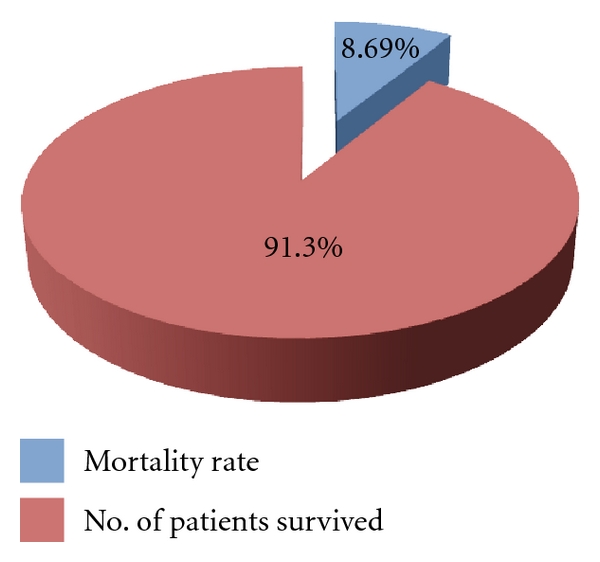
Rate of successful thrombolysis.

All the study population were thrombolysed. Among the 23 of the study population, 2 died with intracerebral hemorrhage, which is an associated risk with the thrombolytic therapy, and when compared to other studies mentioned in our study, it is less. It says that the use of thrombolytic therapy (Alteplase) was safe, and the rate of intracerebral hemorrhage was low (Figure 1).

**Figure 2:**
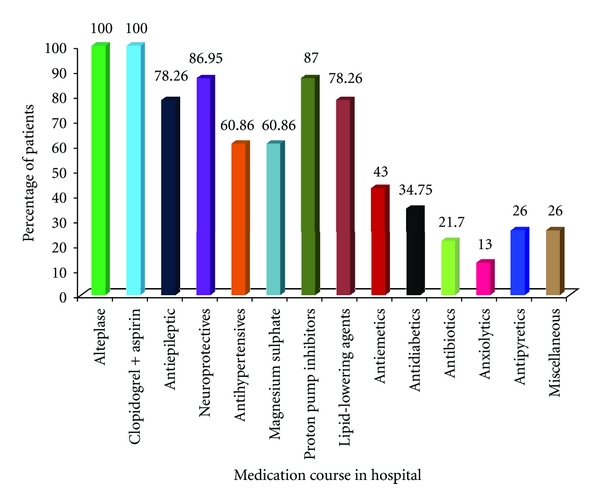
Present medication histories of the patients.

All the study population were treated with the thrombolytic Alteplase (rtPA); clopidogrel and aspirin combination was prescribed for all the study population; neuroprotectives were prescribed for around 87% of the patients, which is followed by lipid-lowering agents and antiepileptics (78.26); anxiolytics was the least prescribed drug in the study population. Proton pump inhibitors were commonly prescribed in 87% of the study population (Figure 2).

Around 39 percent of the study population reached the hospital within 3 hours after the onset of symptoms, and nearly 22% reached between 3 and 4 hours. 13% of the patients reached the hospital within 4 to 5 hours. No patient was found in the time period of above 5 hours, but around 26 percent of patients were not aware of the time of onset of symptoms (Table 3).

The current study revealed that around 61 percentage of the study population were injected the drug Alteplase within 31 to 60 minutes after getting admission to the hospital. The drug was injected initially to thrombolyse the clot and to give speedy recovery to the patient. 22 percent received the treatment within 61 to 90 minutes; only few patients received the treatment after 90 minutes; it indicates that the early treatment with Alteplase is required in most of the patients. None of the study population received the drug immediately once they get in to the hospital or after admission, because the time lag may be used for the initial investigation and diagnosis of the disease (Table 4).

The National Institute of Health Stroke Scale (NIHSS) scores revealed that most of the patients scored high values with the mean of 14.0 when they were admitted in the hospital with a number of disabilities and severities, but after 24 hours, the score was significantly reduced to a mean value of 9.89. This happened because of the effective treatment with the recombinant tissue plasminogen activator (rtPA) ALTEPLASE. A reduction in 4 or more scores after 24 hours clearly shows the effect of drug. This drug was more effective in breaking the clot and allowing the cerebral perfusion. And there was a significant difference in the treatment (Table 5).

The National Institute of Health Stroke Scale (NIHSS) scores revealed that most of the patients scored high values with the mean of 14.0 when they were admitted in the hospital with a number of disabilities and severities, but later, that is, at the time of discharge, the score was remarkably reduced to a mean value of 5.1. This happened because of the effective treatment with the recombinant tissue plasminogen activator (rtPA) ALTEPLASE. This drug was more effective in breaking the clot and allowing the cerebral perfusion. And there is a significant difference in the treatment (Table 6).

mRS score is an outcome score used to predict the outcome of the treatment; the least score is an indicative of good outcome, and higher score is an indicative of poor outcome. In this study, the majority of the patients scored 1 and 2 in mRS scale which indicates that the outcome of the treatment is beneficial and effective. Most of the patients were discharged with very fewer disabilities. In this study population, 2 patients died with the MRS scale score of 6, because of poor recovery (Table 7).

## 4. Conclusion

In this study, most of the patients were male and only one patient was female, which indicates that this stroke problem is more common in male than in female in this locality, but we cannot conclude that thrombolysis is more efficient in male, as it is studied and concluded that there is no difference in the outcome of thrombolysis based on gender [8]. Nearly half of the study population were elderly, which reveals that stroke is mostly affecting the elderly patients; this may be due to several physiological and behavioral reasons; we have also thrombolysed a patient who was above 80 years of age based on the benefit over risk ratio and a study supporting this which concluded that there was a better outcome in patients who were thrombolysed than those who were not thrombolysed; our study comprised of a patient aged more than 80 who was discharged with a desired outcome score [9]. 

 We also found that around 95% of the study population were married. According to this study, smoker alcoholics are having less chance of getting stroke than those not having any type of such habit; in this aspect, our study is more in contrast to various other studies which say that smoking and alcoholism are the major risk factors for stroke. Hypertension and diabetes were the most common diseases we observed in this study population as patient's past medical history; the combination of these diseases in any patient increases the chance of getting stroke, and they are treated with same class of drugs. 

Middle cerebral arteries (MCAs) were mostly affected by stroke, more predominantly left middle cerebral arteries (LT MCA) in the study population than other parts of the brain; this may be due to the degenerative disease of the arteries of brain and high blood pressure. Not only in our study, but also this is common in many other studies like STARS (standard treatment with Alteplase to reverse stroke) [10]. In this study, a large number of patients who were thrombolysed were affected by middle cerebral arteries, more predominantly left, which was similar to our study.

Alteplase is commonly prescribed to thrombolyse the clot for all the patients in our study. Alteplase can also induce intracerebral hemorrhage (ICH), but in our study, only few patients had intracerebral hemorrhage and 2 died due to the same problem; this reveals the safety of the therapy, and this was proven by many studies, one of which is CASES [11]; this study concluded that the rate of ICH is low and use of thrombolytic therapy is safe in actual practice. Most of the patients do not have any bleeding complaint. So this study concludes that Alteplase is safe for the treatment of stroke. 

83 percent of the ages of patients having the previous history of stroke, and now they have admitted in our hospital for the treatment, indicates that the stroke is repeatedly affecting the population and they are in need of proper, safer, and reliable treatment with suitable drugs. Apart from Alteplase, antiplatelets aggregating agents were commonly prescribed in our study population; almost all the patients received the clopidogrel and aspirin combination to lyse the thrombus.

The speed of recovery depends on the arrival of the patients to the hospital after getting the onset of symptoms. Nearly one-third of the patients got admission into the hospital within 3 hours, which is very essential for saving the life of the victim.

Most important is the time for the treatment, and many studies proved that the use of the drug within 3 hours after the stroke symptoms can be very result oriented and safe, a study called (SITS-MOST) [12]; they too concluded that intravenous Alteplase is safe and effective in clinical routine within 3 hours of the symptoms. Drug Alteplase was injected to the patients after 30 minutes of getting admission into this hospital; nearly 60 percent received the drug in the same time period which indicates that the first few minutes were taken to diagnose and investigate the problem.

Our study too came up with a conclusion that thrombolytic therapy is efficacious based on the outcome scores of NIHSS and mRS; our treatment is significant with an NIHSS mean value of prevalue and postvalue after both 24 hours and even during discharge and the mean values of thrombolysis after 24 hours (before 14.0 and after 9.89) were highly significant; a mean discharge score was also obtained (before 14.0 and after 5.1); there were drastic improvements in the scores, and treatment was beneficial as it was studied in STARS [10].

Modified Rankin scale was used to conclude the outcome of the therapy; in our study, we discharged the majority of patients with a score of 0 to 2 which was an expected favorable outcome score to be discharged, and this was similar to CASES [13].

The total sum of the thesis reveals that the use of ALTEPLASE in an acute ischemic stroke is safe and efficacious within a thrombolytic window of 3 to 4.5 hours, provided it should be done in selected population within a well-flourished hospital and well-trained hands with skilled medical teams.

## Figures and Tables

**Table 1:** Past medication history of the study population.

Drugs	Number of patients	Percentage (%)
Antidiabetics	2	8.69
Antihypertensives	3	13.04
Antihypertensives + lipid-lowering therapy	3	13.04
Antianginal + antihypertensives	4	17.39
Antihypertensives + antidiabetics	5	21.73
Antidiabetics + antianginal	1	4.34
Antihypertensives + antiepileptics + antibiotics	1	4.34
Nil	4	17.39

**Table 2:** Area of the brain affected in study population.

Area affected	Number of patients	Percentage (%)
Right middle cerebral arteries (Rt MCAs)	3	13.04
Left middle cerebral arteries (Lt MCAs)	14	60.86
Basilar artery	1	4.3
Intracranial artery	2	8.6
Intracranial artery + middle cerebral arteries	2	8.6
Intracranial artery + middle cerebral arteries + occipital lobe	1	4.3

**Table 3:** Time to reach hospital in the study population.

Time to reach	% of patients
0–3 hr	39.13%
>3-4 hr	21.73%
>4-5 hr	13.04%
>5 hr	0%
Not aware	26.08%

**Table 4:** Door to needle time in study population.

Door to needle time	% of patients
0–30 min	0.00%
31–60 min	60.86%
61–90 min	21.73%
>90 min	4.86%
Not aware	13.04%

**Table 5:** Mean NIHSS of study population after 24 hours.

Nih stroke score	Mean value	*P* value
Before score	14.0	<0.0001
After score	9.89	

**Table 6:** Mean NIHSS of the patient at the time of discharge.

Nih stroke score	Mean value	*P* value
Before socre	14.0	<0.0001
After score	5.1	

**Table 7:** Outcome scores of mRS in percentage.

mRS score	0	1	2	3	4	5	6
% of Patients	13.04%	34.78%	30.43%	8.6%	4.86%	0%	8.6%

## References

[1] Smith WS, Hauser SL, Easton JD, Braunwald E (2001). Cerebrovascular diseases. *Harrison’s Principles of Internal Medicine*.

[2] Zehnder JL, Katzung BG (2007). Drugs used in disorder of coagulation. *Basic and Clinical Pharmacology*.

[3] Smith WS, English JD, Johnson SD, Braunwald D (2008). Cerebrovascular disease. *Harrison’s Principles of Internal Medicine*.

[4] Micieli G, Marcheselli S, Tosi PA (2009). Safety and efficacy of alteplase in the treatment of acute ischemic stroke. *Vascular Health and Risk Management*.

[5] Lacy CF, Armstrong LL, Goldman MP, Lance LL, Lacy CF (2009). Alteplase. *Drug Information Handbook*.

[6] Hill MD, Buchan AM (2005). Thrombolysis for acute ischemic stroke: results of the Canadian alteplase for stroke effectiveness study. *Canadian Medical Association Journal*.

[7] Saver JL (2010). Thrombolytic therapy in stroke. *Emedicine*.

[8] Meseguer E, Mazighi M, Labreuche J, Arnaiz C, Cabrejo L, Slaoui T (2009). Outcomes of intravenous recombinant tissue plasminogen activator according to gender. *Stroke*.

[9] Mishra NK, Ahmed N, Andersen G (2010). Thrombolysis in very elderly people: controlled comparison of SITS international stroke thrombolysis registry and virtual international stroke trials archive. *BMJ*.

[10] Albers GW, Bates VE, Clark WM, Bell R, Verro P, Hamilton SA (2000). Intravenous tissue-type plasminogen activator for treatment of acute stroke: the standard treatment with alteplase to reverse stroke (STARS) study. *Journal of the American Medical Association*.

[11] Ground M, Stenzel C, Schumullling S (1998). Early intravenous thrombolysis for acute ischemic stroke in a community-based approach. *Stroke*.

[12] Wahlgren N, Ahmed N, Davalos A (2008; 08: ). Thrombolysis with alteplase 3–4.5 hours after acute ischemic stroke (SITS—ISTR): an observational study. *The Lancet Neurology*.

[13] Hill MD, Silver FL, Austin PC, Tu JV (2000). Rate of stroke recurrence in patients with primary intracerebral hemorrhage. *Stroke*.

